# Modeling and Compensation of Random Drift of MEMS Gyroscopes Based on Least Squares Support Vector Machine Optimized by Chaotic Particle Swarm Optimization

**DOI:** 10.3390/s17102335

**Published:** 2017-10-13

**Authors:** Haifeng Xing, Bo Hou, Zhihui Lin, Meifeng Guo

**Affiliations:** Engineering Research Center for Navigation Technology, Department of Precision Instruments, Tsinghua University, Beijing 100084, China; xhf15@mails.tsinghua.edu.cn (H.X.); houb15@mails.tsinghua.edu.cn (B.H.); linzh15@mails.tsinghua.edu.cn (Z.L.)

**Keywords:** MEMS gyroscope random drift, phase space reconstruction, back propagation artificial neural network, least squares support vector machine, chaotic particle swarm optimization

## Abstract

MEMS (Micro Electro Mechanical System) gyroscopes have been widely applied to various fields, but MEMS gyroscope random drift has nonlinear and non-stationary characteristics. It has attracted much attention to model and compensate the random drift because it can improve the precision of inertial devices. This paper has proposed to use wavelet filtering to reduce noise in the original data of MEMS gyroscopes, then reconstruct the random drift data with PSR (phase space reconstruction), and establish the model for the reconstructed data by LSSVM (least squares support vector machine), of which the parameters were optimized using CPSO (chaotic particle swarm optimization). Comparing the effect of modeling the MEMS gyroscope random drift with BP-ANN (back propagation artificial neural network) and the proposed method, the results showed that the latter had a better prediction accuracy. Using the compensation of three groups of MEMS gyroscope random drift data, the standard deviation of three groups of experimental data dropped from 0.00354°/s, 0.00412°/s, and 0.00328°/s to 0.00065°/s, 0.00072°/s and 0.00061°/s, respectively, which demonstrated that the proposed method can reduce the influence of MEMS gyroscope random drift and verified the effectiveness of this method for modeling MEMS gyroscope random drift.

## 1. Introduction

MEMS (Micro Electro Mechanical System) gyroscopes have the advantages of being small in size, lightweight, low cost, vibration resistant, and so on. Therefore, MEMS gyroscopes have been used widely in civil and military fields such as automobiles, UAVs (unmanned aerial vehicles) and weapon guidance systems [1,2]. However, compared to high precision laser gyroscopes or fiber optic gyroscopes, the accuracy of the MEMS gyroscope is low and the random drift has nonlinear and non-stationary characteristics due to the limitation of the current material processing technology [3,4,5]. These disadvantages make the MEMS gyroscopes still inapplicable in many high-precision fields. Therefore, it is of great significance for modeling to compensate for MEMS gyroscope random drift.

Presently, the common methods for modeling MEMS gyroscope random drift are two classes, one is the statistical modeling method represented by traditional time series analysis, and the other is the intelligence algorithm represented by ANNs (artificial neural networks) for modeling the MEMS gyroscope random drift [6,7,8]. The time series model of random drift assumes that MEMS gyroscope measurement noise is a linear combination of historical data and historical white noise, and the ARMA (auto regressive and moving average) model for drift modeling is often used [9]. However, this method assumes the random drift is stationary which will inevitably limit the range of application and the prediction accuracy of the model [3]. Some scholars put forward reducing the adverse effect of the MEMS gyroscope random drift using a wavelet analysis method, since wavelet transform can subdivide the specified signal in the frequency domain and time domain simultaneously to obtain more details. Conversely, Fourier transform can only be used in the frequency domain analysis, so wavelet transform has a better signal processing ability [10,11].

Through the development of intelligent algorithms, there are more ways to model and compensate the random drift of MEMS gyroscope. The ANNs are common intelligent algorithms, and have achieved good results in random drift modeling [12,13,14]. While ANNs can theoretically approximate any nonlinear function, there may be overfitting problems during training, and the number of nodes in the hidden layer depends on experience and lacks theoretical guidance [15,16]. The LSSVM (least squares support vector machine) proposed by Suyken is one of the most important achievements of statistical learning theory; compared with the standard SVM (support vector machine) algorithm, the LSSVM follows the same principle of SRM (structural risk minimization) and has the advantage of reducing the computational complexity, improving the calculation speed and the anti-interference ability; compared to the ANNs that have attracted much attention in the nonlinear field, this algorithm is not prone to fall into local optimization and has better generalization performance, especially if there are not enough learning samples [17,18,19]. Some scholars had proposed to use the LSSVM to predict MEMS gyroscope random drift [20,21]. However, too many data were used according to the collection order for prediction, and there was no particularly clear data construction standard and the dimension of the input vector was high, thus leading to computational complexity.

This paper proposes to process the raw data of MEMS gyroscopes with wavelet filtering and PSR (phase space reconstruction), then model the reconstructed data based on LSSVM, and using CPSO (chaotic particle swarm optimization) to optimize the parameters of LSSVM. This algorithm can be abbreviated as CPSO-LSSVM. To the best of the authors’ knowledge, it is the first report on the combination of PSR and CPSO-LSSVM for modeling and compensating the random drift of MEMS gyroscope.

The purpose of this paper is to establish a more accurate MEMS gyroscope random drift model based on the CPSO-LSSVM method; to do so, the steps of building the model are described in detail, including dealing with the original data, reconstructing the data and analyzing and modeling the reconstructed data. The comparison with BP-ANN (back propagation artificial neural network) has verified that this algorithm has a better effect.

The structure of this paper is organized as follows: The principle of BP-ANN and the proposed method, that is, CPSO-LSSVM, are clearly described in Section 2; the steps of constructing an MEMS gyro drift model are discussed in Section 3; experiments and results of BP-ANN and CPSO-LSSVM for modeling the gyroscope random drift are illustrated in Section 4. Finally, Section 5 draws the conclusion and ends the paper.

## 2. The Principles of Algorithms

Artificial neural networks, ANNs, and LSSVMs (least square support vector machines) are two kinds of machine learning algorithms that can achieve nonlinear mapping. Both ANNs and LSSVMs have been applied to analyze data that are not mathematically modeled easily. The principles of two algorithms are described in this section.

### 2.1. Back Propagation Artificial Neural Networks

Artificial neural networks (ANNs) simulating biological neural systems have a powerful learning function. The weighted sum of inputs arriving at each neuron generates an output signal by means of an activation function [22,23,24]. The BP-ANN (back propagation artificial neural network) is one of the most employed ANN methods and has been widely used in many applications. It does not require a rigorous mathematic model and can obtain calibration parameters for data through a learning step [25]. Good flexibility makes it widely used to express nonlinear relationships in databases [26].

**Figure 1:**
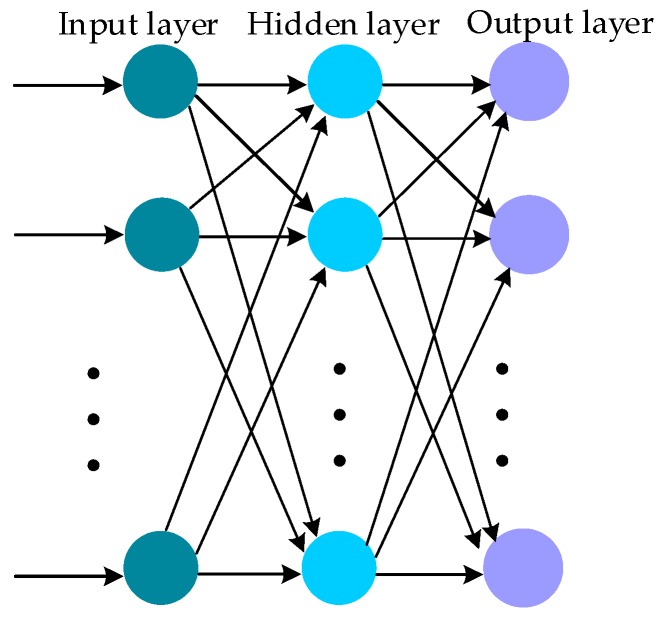
The classical architecture of BP-ANN (back propagation artificial neural network).

The BP-ANN consists of three types of layers which are the input layer, hidden layer and output layer, and its classical architecture is revealed in Figure 1. The nodes of input layer are used to select independent variables for estimation. The hidden layer is important and has an impact on the learning ability of the BP-ANN. The output layer is used to estimate the result depending on the independent input variables.

The input of neurons in the hidden layer can be described as:

(1) $$v_{i}=\sum_{i=1}^{n_{0}}w_{ij}x_{j}+b_{i}$$

where $v_{i}$ is the ith neuron input in the hidden layer, $w_{ij}$ is the connection weight between the ith neuron in the input layer and the jth neuron in the hidden layer, $x_{j}$ denotes the jth neuron input in the input layer while $n_{0}$ is the number of neurons in the input layer, and $b_{i}$ is the threshold of the ith neuron in the hidden layer.

The sigmoid function presented in (2) is the activation function of the hidden layer:

(2) $$g\left(x\right)=\frac{1}{1+e^{-x}}$$

where *x* is the independent variable.

The output of the neuron in the hidden layer can be formulated as (3) with the sigmoid function shown in (2):

(3) $$x_{i}=g\left(v_{i}\right)$$

where $x_{i}$ is the ith neuron output in the hidden layer, and $v_{i}$ is the same as for (1).

The activation function in the output layer is the same as that in the hidden layer, and the weight and threshold of the neuron in the output layer are also constantly adjusted to approximate the desired output value.

**Figure 2:**
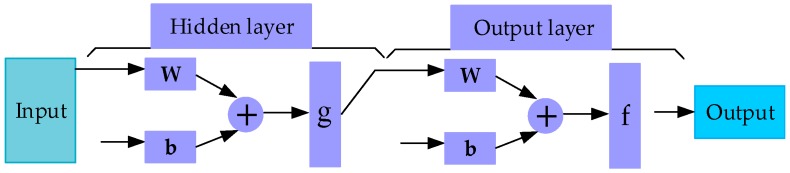
The schematic diagram of the BP-ANN.

Figure 2 is the scheme diagram of the BP-ANN illustrating the neural network architecture, where W is the connection weight of each node, *b* is a threshold, and *g* and *f* are the activation function shown in (2).

However, it must be pointed out that ANNs follow the principle of ERM (empirical risk minimization) and might result in over-fit toward the employed dataset [27]. In particular, when the amount of data is not too large, some algorithms must be applied to improve ANNs or other machine learning algorithms should be considered to avoid over-fit.

### 2.2. LSSVM Model with CPSO

**Figure 3:**
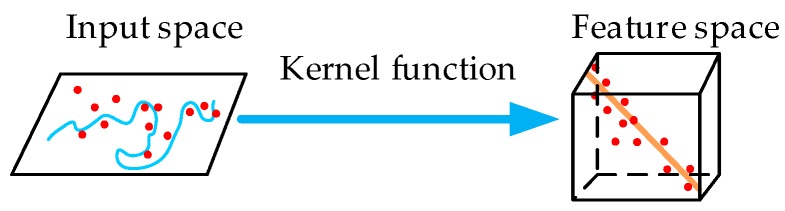
The principle of LSSVMs (least squares support vector machine).

The LSSVM, one of the new soft computing learning algorithms, was developed by Suyken [17]. The LSSVM is performed by solving a linear system of equations instead of quadratic programming as for the standard SVM case [28]. Since LSSVMs have SRM of the standard SVMs and a faster computation speed, LSSVMs have been successfully applied to pattern recognition, fault diagnosis and function estimation [29,30,31]. The principle of LSSVMs is shown in Figure 3.

Suppose a training dataset ${\left\{x_{i}\;y_{i}\right\}}_{i}^{n}$, where $x_{i}$ is the *n*-dimensional input vector, $x_{i}\in$ $R_{n}$, and $y_{i}$ is the responding desired output, $y_{i}\in R$. The training data can fit with the function represented as:

(4) f(x)=w·∅(x)+b

where ∅(x) is a nonlinear mapping between input space x and high-dimensional feature space, w denotes a weight vector, and *b* denotes a constant offset.

The w and *b* can be estimated through minimization of regularized risk function subjected to the equality constraint shown in (5):

$$\mathrm{Minimize}\;J\left(w,\;e\right)=\frac{1}{2}||w^{2}||+\frac{1}{2}\gamma \sum_{i=1}^{n}\left(e_{i}^{2}\right)$$

(5) $$\mathrm{Subject}\;\mathrm{to}\;y_{i}\left[w^{T}\emptyset \left(x_{i}\right)+b\right]=1-e_{i},\;i=1,2,\ldots n$$

where $e_{i}$ is the introduced error variable, and γ is an adjustable parameter.

The corresponding Lagrange for Equation (5) is:

(6) $$L\left(w,b,e;a\right)=J\left(w,\;e\right)-\sum_{i=1}^{n}a_{i}\left\{y_{i}\left[w^{T}\emptyset \left(x_{i}\right)+b\right]-1+e_{i}\right\}$$

The optimality condition of Equation (6) leads to the following linear system:

(7) $$\left[\begin{array}{cc} 0 & y^{T}\\ y & \Omega +I/y \end{array}\right]\left[\begin{array}{c} b\\ a \end{array}\right]=\left[\begin{array}{c} 0\\ I \end{array}\right]$$

where $\Omega_{ij}=\Phi \left(x_{i},x_{j}\right)$ is the kernel function and has optional kernels such as Gaussian kernel, polynomial kernel, B-spine kernel, and so on. A Gaussian kernel is usually chosen due to its simple, efficient and reliable computing power [32]. The Gaussian kernel can be written as follows:

(8) $$\Phi \left(x_{i},x_{j}\right)=\exp\left(-\frac{{\left||x_{i}-x_{j}|\right|}^{2}}{\sigma^{2}}\right)$$

where $\sigma^{2}$ represents the kernel parameter, and $x_{i}$ and $x_{j}$ are two independent vectors in the input space.

However, the LSSVM’s parameters are uncertain for different problems and must be carefully determined because this will affect the modeling effect. It was proposed to use CPSO (chaotic particle swarm optimization) to optimize the two parameters of LSSVM. What needs to be noted is that CPSO is an improved algorithm for PSO (particle swarm optimization). Similar to GA (genetic algorithms) and evolutionary programming, PSO is also a heuristic searching method, but it does not contain complex mechanisms such as crossover or mutation [33]. The PSO algorithm is fast and suitable for parallel computing. Since chaotic mapping has deterministic, ergodic and stochastic properties, chaos mapping is introduced into PSO, which is called CPSO [34,35].

During calculation, the particle moving in space is influenced by three factors, including the particle’s current velocity v(*t*), the best point $p_{id}$ where the particle has arrived before and optimum point of the community $p_{gd}$. Concurrently, the three factors are respectively assigned to a random weight. The velocity and position are updated on the basis of Equations (9) and (10):

(9) $$v_{id}\left(t+1\right)=\omega \times v_{id}\left(t\right)+c_{1}\times r_{1}\times \left(p_{id}-z_{id}\right)+c_{2}\times r_{2}\times \left(p_{gd}-z_{id}\right)$$

(10) $$z_{id}\left(t+1\right)=z_{id}\left(t\right)+v_{id}\left(t+1\right)$$

(i=1,2,…,n d=1,2,…,m)

where *c*_1_ and *c*_2_ denote the learning factors, usually, *c*_1_ = *c*_2_ = 2; ω is the weight; *r*_1_, *r*_2_ are the random numbers within [0, 1]; z denotes the current position of the particle; t is the number of iterations; n and *m*, respectively, represent the number of particles and the dimension of the particle.

The CPSO process can be described as follows:

Step 1. Chaos Initialization: A random vector defined as $z=\left[z_{1},z_{2},\ldots ,z_{D}\right]$ is generated, *D* denotes the dimension of the variable that needs to be optimized. The range of each component of z is [0, 1]. Then, *M* components are obtained according to Equation (11).

(11) $$z_{n+1}=\mu \times z_{n}\times \left(1-z_{n}\right)\;n=0,1,2,\ldots \;\mathrm{Subject}\;\mathrm{to}\;\{\begin{array}{c} \mu \in \left[0,4\right]\\ 0<z_{n}<1 \end{array}$$

The chaotic interval is mapped to the range of variables based on Equation (12):

(12) $$x_{ij}=a_{j}+\left(b_{j}-a_{j}\right)\times z_{ij}\;(i=1,2\ldots ,M;\;j=1,2\ldots ,D)$$

where $b_{j}$ and $a_{j}$ are the upper and lower limits of the optimization variables, respectively.

Then, calculating the fitness value of each particle according to the objective function shown in Equation (13):

(13) $$f_{objective}=\frac{1}{n}\overset{n}{\underset{i=1}{\sum }}\left|y_{i}-\hat{y_{i}}\right|$$

where *n* represents the sample size of the training dataset. The output training set is $y_{i}$ and ${\hat{y}}_{i}$ is the corresponding fitting result of $y_{i}$.

The *N* particles with better performance are selected as the initial solution from the initial particle swarm with *M* particles, and the particle velocity is generated at random.

Defining the current position of the particle is individual best, then calculating the corresponding fitness value and setting the position of the particle whose fitness value is the best is the global best point. The global best corresponds to the minimum fitness value.

Step 2. Velocity and Position Updating: The velocity and position of ith particle in the dth dimension is updated according to Equations (9) and (10), with the global best and individual best. The parameter ω is updated by the Equation (14):

(14) $$\omega \left(t\right)=\omega_{max}-t\times \frac{\omega_{max}-\omega_{min}}{t_{max}}$$

where $\omega_{max}$ and $\omega_{min}$ are the maximum and minimum values of initial weights which can be set as 0.9 and 0.1, respectively, t denotes the current number of iterations, while $t_{max}$ is the maximum number of iterations set.

Step 3. Individual and Global Best Updating: Calculating the fitness value of each particle and evaluating this particle according to its updated position. When the fitness value of this particle is less than that of individual or global best, then individual or global best will be replaced by the position of this particle.

Step 4. Stopping Criteria: If one stopping condition is satisfied then stop; otherwise go to Step 2.

**Figure 4:**
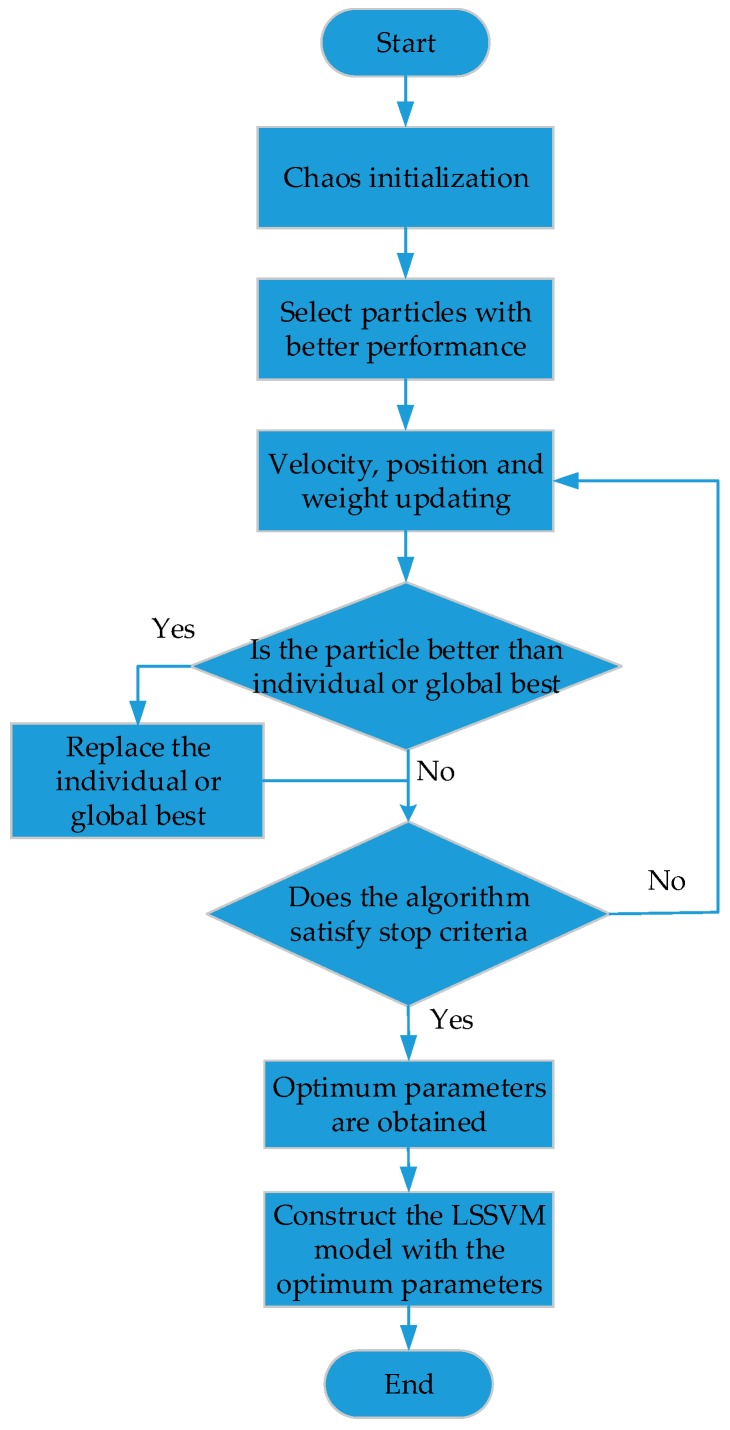
The flow chart of CPSO (chaotic particle swarm optimization)-LSSVM.

Based on descriptions above, the flow chart of CPSO-LSSVM is shown in Figure 4:

## 3. Construction of MEMS Gyroscope Drift Model

**Figure 5:**
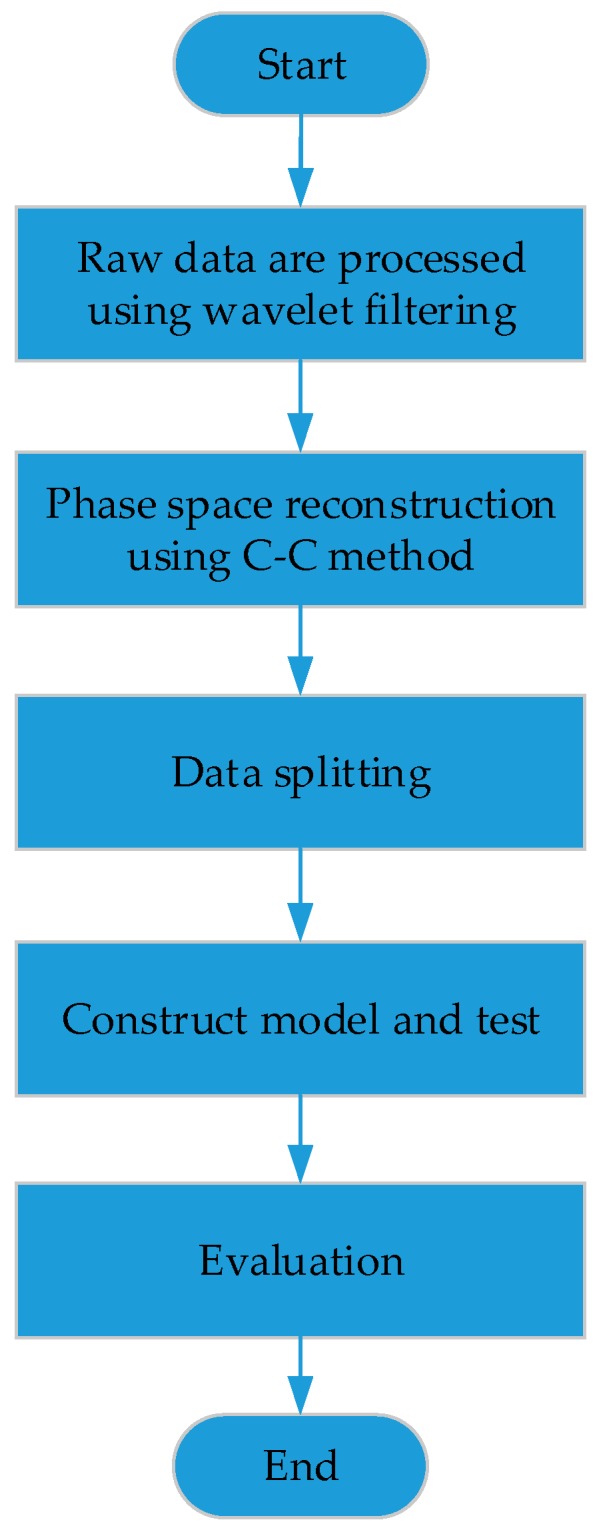
The flow chart of constructing a MEMS (Micro Electro Mechanical System) gyroscope model.

The CPSO-LSSVM method is used to model the MEMS gyroscope random drift, and is compared with BP-ANN, which is widely used in nonlinear fields. The original MEMS gyroscope data unavoidably includes noise information. Therefore, the wavelet filtering method is used to eliminate noise, and the random drift of the MEMS gyroscope is obtained for constructing a model. The random drift is a chaotic time series, and it is reconstructed by PSR since it is an efficient algorithm to analyze chaotic time series; the dimension of the random time series is improved by embedding the one-dimensional time series into an auxiliary phase space, thus improving the prediction accuracy [3,36]. Then, the reconstructed data is modeled and analyzed. Details of the model are expressed as follows, and the main procedures are illustrated as follows and depicted in Figure 5:

Step 1: Wavelet Filtering. The wavelet analysis overcomes the shortcomings of Fourier analysis in signal processing and can remove the noise while preserving the signal instantaneous dynamic characteristics, so it has a wide range of applications in signal de-noising [37,38]. The original MEMS gyroscope drift contained noise components, and the mother wavelet ’db4’ and the soft threshold are adopted for de-noising. 

Step 2: Phase Space Reconstruction. Regarding the random time series: {x(i)}, *i* = 1, 2,..., *N*, the reconstructed sequence is obtained through PSR method as follows:

(15) $$X=\left[\begin{array}{ccc} \begin{array}{c} \begin{array}{cc} x_{1} & x_{1+\tau } \end{array}\\ \begin{array}{cc} x_{2} & x_{2+\tau } \end{array} \end{array} & \cdots & \begin{array}{c} x_{1+\left(m-1\right)\tau }\\ x_{2+\left(m-1\right)\tau } \end{array}\\ \vdots & \ddots & \vdots \\ \begin{array}{cc} x_{n-\left(m-1\right)\tau -1} & x_{n-\left(m-2\right)\tau -1} \end{array} & \cdots & x_{n-1} \end{array}\right]$$

(16) $$Y={\left[\begin{array}{ccc} x_{2+\left(m-1\right)\tau } & x_{3+\left(m-1\right)\tau } & \begin{array}{cc} \cdots & x_{n} \end{array} \end{array}\right]}^{T}$$

where m represents embedding dimension and τ denotes delay time.

The delay time window, $\tau_{w},$ shown in Equation (17) is introduced as follows:

(17) $$\tau_{w}=\left(m-1\right)\tau$$

The C-C method is used to determine optimal parameters $\tau_{w}$ and τ since this method is robust, relatively simple and not computationally demanding [39]. The following three formulas are very important for determining the parameters:

(18) $$\bar{S}\left(t\right)=\frac{1}{16}\overset{5}{\underset{m=2}{\sum }}\overset{4}{\underset{j=1}{\sum }}S\left(m,r_{j},t\right)$$

(19) $$\Delta \bar{S}\left(t\right)=\frac{1}{4}\overset{5}{\underset{m=2}{\sum }}\Delta S\left(m,t\right)$$

(20) $$S_{cor}\left(t\right)=\Delta \bar{S}\left(t\right)+\left|\bar{S}\left(t\right)\right|$$

where *m* = 2,3,4,5, $r_{j}=\frac{i\sigma }{2}$, *i* = 1,2,3,4 and $S\left(m,r_{j},t\right)$ and ΔS(m,t) are two functions whose definitions have been elaborated in the paper [39]. 

Looking for the first zero point of $\bar{S}\left(t\right)$ or the first local minimum of $\Delta \bar{S}\left(t\right)$ to find the first locally optimal time for independence of the data gives the delay time τ. Meanwhile, by simply looking for the minimum of $S_{cor}\left(t\right)$ and then the delay time window, $\tau_{w}$ can be obtained [37,39].

Step 3: Data Splitting. After the noise removal and data reconstruction, the MEMS gyroscope random drift sequence is split into the training dataset and the testing dataset. To eliminate the impact of amplitude and improve convergence performance, data normalization is needed to be done, based on Equation (21) as follows:

(21) $$x_{k}^{\prime }=\frac{\left(x_{\max}^{\prime }-x_{min}^{\prime }\right)\left(x_{k}-x_{min}\right)}{x_{max}-x_{min}}$$

where $x_{max}$ is the maximum value of the variable and $x_{min}$ represents the minimum value$,\;\mathrm{with}\;x_{max}^{\prime }$ set as 1 and $x_{min}^{\prime }$ is set as −1. The range of variables is normalized within [−1, 1] after preprocessing.

Step 4: Model Construction and Test. This step requires a training dataset to train the model, and uses a testing dataset to verify the prediction accuracy of the proposed model. The algorithms of different models have been described in detail in Section 2 of this paper.

Step 5: Evaluation. The prediction error indexes, namely MAE (mean absolute error), RMSE (root mean square error), and ARE (average relative error), are applied for evaluating prediction accuracy. The application of these indices is very common, so are only briefly described here:

(22) $$MAE=\frac{1}{n}\overset{n}{\underset{i=1}{\sum }}\left|y_{pi}-y_{i}\right|$$

(23) $$RMSE=\sqrt{\frac{\sum_{i=1}^{n}{\left(y_{pi}-y_{i}\right)}^{2}}{n}}$$

(24) $$\mathrm{ARE}=\frac{1}{n}\overset{n}{\underset{i=1}{\sum }}\frac{\left|y_{pi}-y_{i}\right|}{y_{i}}$$

where n is the size of prediction data, $y_{pi}$ denotes the predicting value, and $y_{i}$ represents the actual value. 

## 4. Results and Discussion

### 4.1. Experiment Setup 

**Figure 6:**
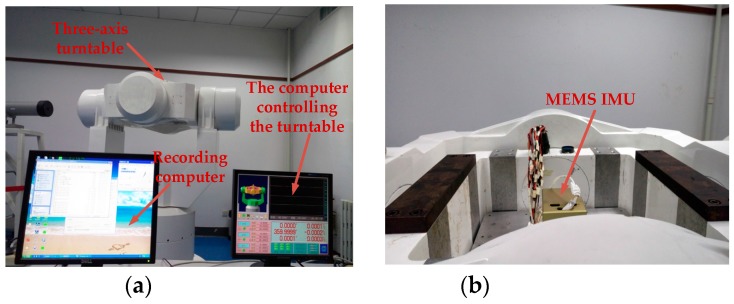
(**a**) Experimental environment; (**b**) the MEMS IMU (inertial measurement unit) used in the experiment.

The hardware utilized in the trials was an MEMS IMU (inertial measurement unit) designed by this laboratory. The MEMS IMU was placed on the three-axis turntable. Preheating the MEMS IMU lasted for 20 min, then 1500 s of data were collected, and the sampling rate was 10 Hz. The output data of the X-axis gyroscope were analyzed and modeled in this paper. The experiment environment and MEMS IMU are shown in Figure 6.

### 4.2. Data Preprocessing

**Figure 7:**
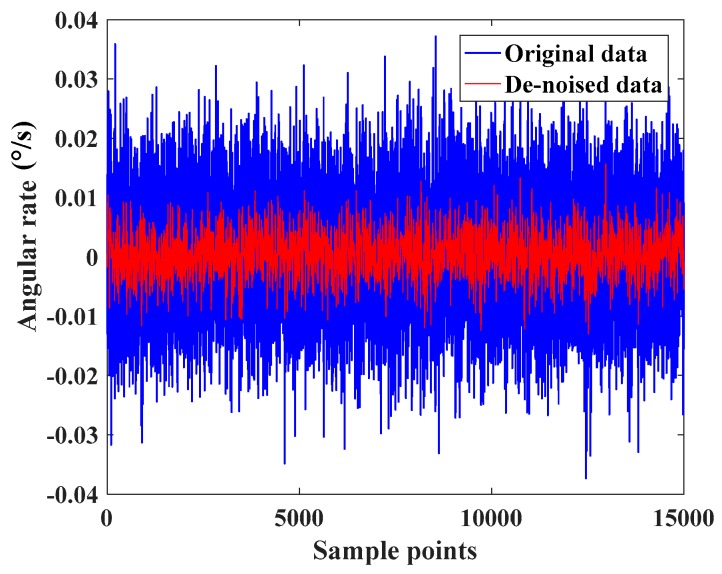
Original data and de-noised data of the MEMS gyroscope.

Section 3 notes the task of data preprocessing was to perform wavelet filtering and phase space reconstruction for the collected data of MEMS gyroscope. Following deterministic error compensation, the time series of random drift was obtained. First wavelet filtering with mother wavelet ‘db4’ and the soft threshold was used to remove most of the noise of original data under three scales. Figure 7 shows the de-noised data contained the essential features of random drift. 

The next step was to reconstruct the filtered series. The C-C method was used to determine the delay time τ and the time window $\tau_{w}$. The related equations are presented as Equations (18)–(20).

**Figure 8:**
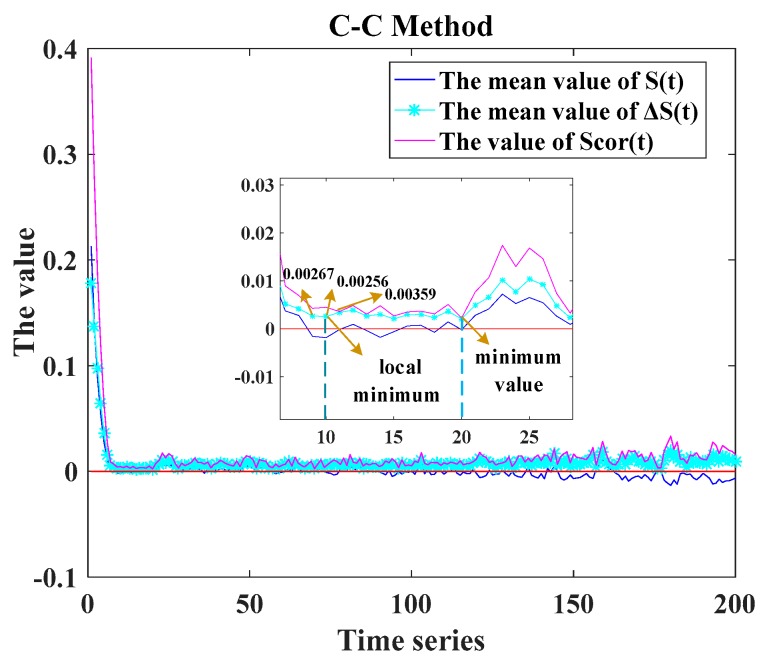
Determining the delay time and the time window by using the C-C method.

Figure 8 shows the time delay τ corresponding to the first local minimum of $\bar{\Delta S}\left(t\right)$ was 10. The time window $\tau_{w}$ corresponding to the minimum value of $S_{cor}\left(t\right)$ was 20. According to the Equation (17), m=3 was easily obtained. Then, the reconstructed mathematical matrixes were obtained as follows:

(25) $$X=\left[\begin{array}{ccc} x_{1} & x_{11} & x_{21}\\ x_{2} & x_{12} & x_{22}\\ \begin{array}{c} \vdots \\ x_{n-21} \end{array} & \begin{array}{c} \vdots \\ x_{n-11} \end{array} & \begin{array}{c} \vdots \\ x_{n-1} \end{array} \end{array}\right]$$

(26) $$Y={\left[\begin{array}{ccc} x_{22} & x_{23} & \begin{array}{cc} \ldots & x_{n} \end{array} \end{array}\right]}^{T}$$

### 4.3. Comparing the Effects of Modeling 

To verify the applicability of the proposed model, the reconstructed data (25) and (26) were normalized by using Equation (21) at first, then the data were divided into three groups. Each group then was divided into two data sets: the training dataset (80%), and the testing dataset (20%). Furthermore, the training set was further divided into an input-training set and an output-training set. Detailed descriptions were as follows.

**Figure 9:**
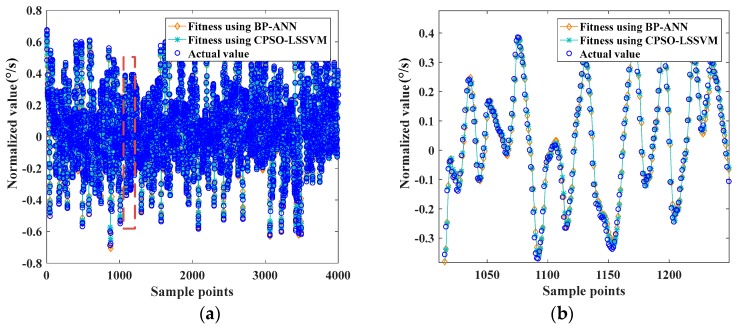
(**a**) The fitting effect of the training dataset using BP-ANN and CPSO-LSSVM; (**b**) partially enlarged detail of (**a**).

The first group containing 5000 sets of data was used for establishment and validation of BP-ANN and CPSO-LSSVM models at first. The training dataset contained 4000 sample points, and the other 1000 sample points were for testing. The BP-ANN method, with 10 neurons in hidden layer, was used for the training dataset. The parameters of LSSVM, which had been acquired on the basis of CPSO, were $\gamma =29.358,\;\sigma^{2}$ = 83.162. The fitting effect of the training dataset was shown in Figure 9.

**Figure 10:**
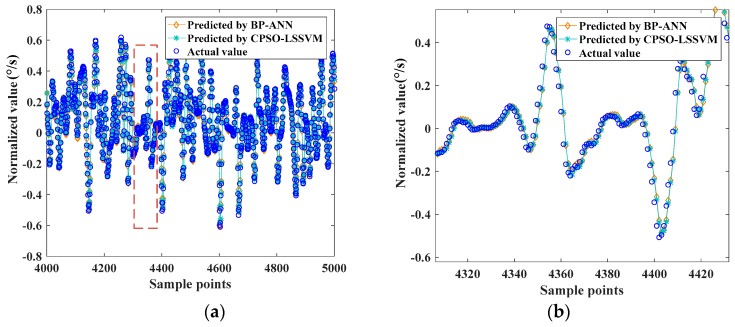
(**a**) The effect of prediction of the testing dataset using BP-ANN and CPSO-LSSVM; (**b**) partially enlarged detail of (**a**).

The two well-trained models were used to predict the output of the testing dataset, and the predicted values were compared with the actual values, which are shown in Figure 10:

Statistical parameters, such as MAE, RMSE and ARE, depicted in Equations (22)–(24), were used to evaluate the prediction accuracy. Table 1 depicts the statistical analysis of the comparison results of the two models.

Figure 9 and Figure 10 show that BP-ANN and CPSO-LSSVM both had a good effect on fitting the random drift, but the CPSO-LSSVM was better according to Table 1. The training time of BP-ANN was about 22.301 s, while the CPSO-LSSVM was about 14.556 s in the first group, so the latter had a better computing speed. It further demonstrated the applicability of the proposed method.

**Figure 11:**
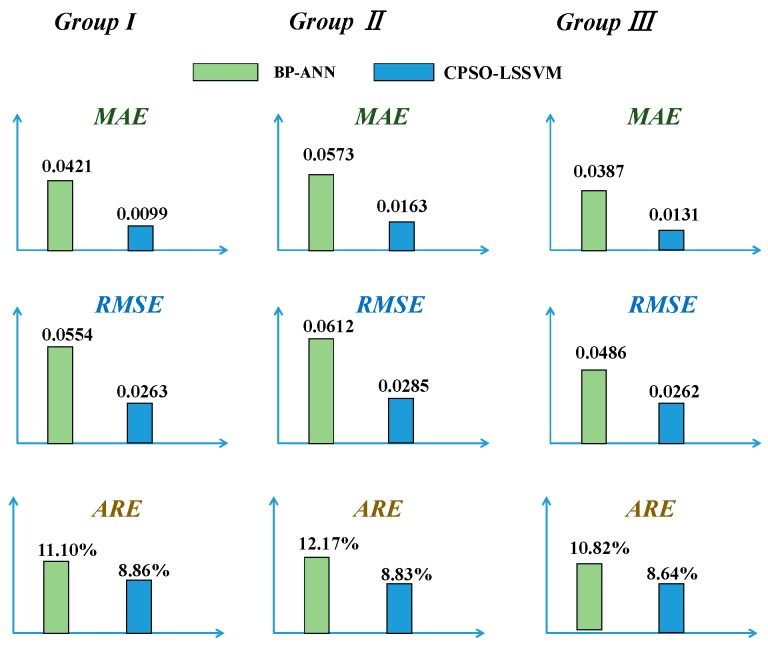
The statistical results of the three groups.

Furthermore, the reproducibility of both methods was evaluated by building models for the other two groups. The results of the three groups were vividly presented in Figure 11. Comparing the prediction results of the other two groups reaches conclusions similar to that which the first group obtained.

Based on the results above, the proposed CPSO-LSSVM method outperforms the BP-ANN. The better prediction performance may be due to the tendency for the CPSO to enjoy certainty, ergodicity and stochastic property, and to have a good global search capability; LSSVSM has the characteristics of SRM and anti-interference ability; CPSO-LSSVM combines both the advantages of CPSO and LSSVM.

**Figure 12:**
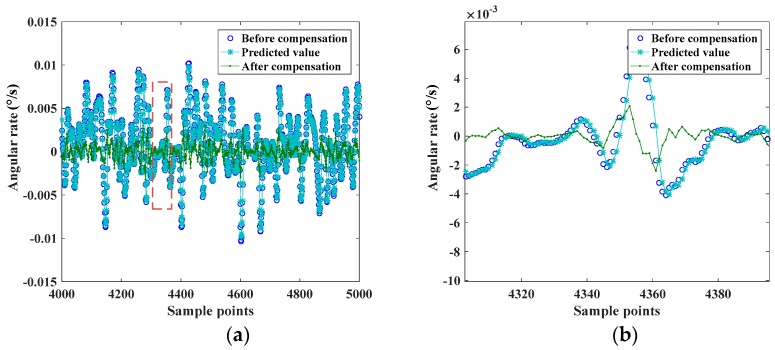
(**a**) The change of random drift before and after compensation; (**b**) partially enlarged detail of (**a**).

The compensation of MEMS gyroscope drift then was done based on the prediction values of CPSO-LSSVM method. The anti-normalization of prediction data were used to compensate the random drift of MEMS gyroscope. Comparing the testing data in the first group before and after the compensation, the results showed that the compensative effect was remarkably effective. The standard deviation of the random drift after compensation was 0.00065°/s, while it was 0.00354°/s without compensation. Figure 12 shows the random drift of the MEMS gyroscope before and after the compensation.

The effect of compensating the random drift using CPSO-LSSVM method for the three groups was shown in Table 2, where the evaluation index was the standard deviation of the random drift.

Observing Table 2, it was clear that the standard deviation of the random drift had been decreased significantly after compensation. The results further demonstrated the applicability and validity of the CPSO-LSSVM method. This method was a feasible and satisfactory way to establish the model for MEMS gyroscope random drift.

## 5. Conclusions

The modeling of MEMS gyroscope random drift is a hot research topic since an accurate model is beneficial to improve the accuracy of MEMS gyroscopes. The key contribution of this paper is to reconstruct the random drift data of the MEMS gyroscope with PSR using the C-C method, and then to analyze the reconstructed data by the BP-ANN and CPSO-LSSVM methods. Based on the results and analyses, the following conclusions can be drawn: (a) Through a comparison of the results from the above two analysis methods, the statistical indicators, including MAE, RMSE, and ARE, for the testing dataset indicate that the proposed method has a better prediction precision; (b) the availability and effectiveness of CPSO-LSSVM method are demonstrated by an obvious decrease in the standard deviation of the random drift after compensation, which is shown in Table 2; (c) PSR plays an important role in the modeling of MEMS gyroscope random drift since it can reduce the dimension of input vector during modeling, thus reducing computation cost and complexity; (d) the CPSO can perform more powerful searching capability for parameters to construct the proposed model, and it can also be used for function optimization, data mining, and so on; (e) the combination and improvement of intelligent algorithms can lead to a better algorithm, and the CPSO-LSSVM method presented in this paper validates this point; (f) whether the modeling accuracy and compensation effectiveness of the method are affected by the different sensing mechanisms should be further studied. Considering using an algorithm to compensate for random drift of MEMS gyroscope is not enough, as well as from the structure and design standpoint to improve the accuracy of the MEMS gyroscope.

To summarize, the results show the better prediction capacity of the proposed model. It is believed that this algorithm can be regarded as a reliable method for modeling and compensating the MEMS gyroscope random drift. This method is expected to be applied in the field of north-seeking and short-time navigation based on MEMS gyroscopes. Additionally, the method proposed in this paper also implies that this method can be a new way for pedestrian step estimation, pattern recognition and many other fields. Better algorithms and more methods need to be studied further, and more work needs to be done in the future.

## Figures and Tables

**Table 1:** The statistical analysis of BP-ANN and CPSO-LSSVM. MAE (mean absolute error); RMSE (root mean square error); ARE (average relative error).

Model	MAE (°/s)	RMSE (°/s)	ARE
BP-ANN	0.0421	0.0554	11.10%
CPSO-LSSVM	0.0099	0.0263	8.86%

**Table 2:** The standard deviation of the random drift before and after compensation.

Group	I	II	III
Before compensation (°/s)	0.00354	0.00412	0.00328
After compensation (°/s)	0.00065	0.00072	0.00053

## References

[1] Wang X.Y., Meng X.Y. Research on Time-series Modeling and Filtering Methods for MEMS Gyroscope Random Drift Error. Proceedings of the Materials Science and Engineering Conference Series.

[2] Li B.-W., Yao D.-Y. (2014). Low-cost MEMS IMU navigation positioning method for land vehicle. J. Chin. Inert. Technol..

[3] Wang S., Deng Z., Yin G. (2016). An Accurate GPS-IMU/DR Data Fusion Method for Driverless Car Based on a Set of Predictive Models and Grid Constraints. Sensors.

[4] Chia J., Low K., Goh S., Xing Y. A low complexity Kalman filter for improving MEMS based gyroscope performance. Proceedings of the Aerospace Conference.

[5] Cao H., Li H., Kou Z., Shi Y., Tang J., Ma Z., Shen C., Liu J. (2016). Optimization and experimentation of dual-mass MEMS gyroscope quadrature error correction methods. Sensors.

[6] Hsu Y.L., Chou P.H., Kuo Y.C. Drift modeling and compensation for MEMS-based gyroscope using a Wiener-type recurrent neural network. Proceedings of the IEEE International Symposium on Inertial Sensors and Systems.

[7] Liu Y., Niu R., Wang C., Wang L. (2013). Random Error Model and Compensation of MEMS Gyroscope Based on BP Neural Network. Int. J. Digit. Content Technol. Its Appl..

[8] Ansari A., Bakar A.A. A Comparative Study of Three Artificial Intelligence Techniques: Genetic Algorithm, Neural Network, and Fuzzy Logic, on Scheduling Problem. Proceedings of the Artificial Intelligence with Applications in Engineering and Technology (ICAIET).

[9] Chen M.M., Gao G.W. (2014). Research on MEMS gyroscope random error compensation algorithm based on ARMA model. Appl. Mech. Mater..

[10] Yuan J., Yuan Y., Liu F., Pang Y., Lin J. (2015). An improved noise reduction algorithm based on wavelet transformation for MEMS gyroscope. Front. Optoelectron..

[11] Shi Y.S., Gao Z.F. (2013). Study on MEMS gyro signal de-noising based on improved wavelet threshold method. Appl. Mech. Mater..

[12] Zha F., Xu J., Li J., He H. (2013). IUKF neural network modeling for FOG temperature drift. J. Syst. Eng. Electron..

[13] Chong S., Rui S., Jie L., Xiaoming Z., Jun T., Yunbo S., Jun L., Huiliang C. (2016). Temperature drift modeling of MEMS gyroscope based on genetic-Elman neural network. Mech. Syst. Signal Process..

[14] Xu D., Yang Z., Zhao H., Zhou X. A temperature compensation method for MEMS accelerometer based on LM_BP neural network. Proceedings of the 2016 IEEE Sensors.

[15] Fei J., Wu D. (2017). Adaptive control of MEMS gyroscope using fully tuned RBF neural network. Neural Comput. Appl..

[16] Deng F., Guo S., Zhou R., Chen J. (2017). Sensor multifault diagnosis with improved support vector machines. Trans. Autom. Sci. Eng..

[17] Suykens J.A., Vandewalle J. (1999). Least squares support vector machine classifiers. Neural Process. Lett..

[18] You H., Ma Z., Tang Y., Wang Y., Yan J., Ni M., Cen K., Huang Q. (2017). Comparison of ANN (MLP), ANFIS, SVM, and RF models for the online classification of heating value of burning municipal solid waste in circulating fluidized bed incinerators. Waste Manag..

[19] Sun T., Liu J. Predicting MEMS gyroscope’s random drifts using LSSVM optimized by modified PSO. Proceedings of the Guidance, Navigation and Control Conference (CGNCC).

[20] Bo R., Huan L. On forecast modeling of MEMS gyroscope random drift error. Proceedings of the 2015 34th Chinese Control Conference (CCC).

[21] Qin W.-W., Zheng Z.-Q., Liu G., Wang L.-X. (2008). Modeling method of gyroscope's random drift based on wavelet analysis and LSSVM. J. Chin. Inertial Technol..

[22] Torrecilla J.S., Deetlefs M., Seddon K.R., Rodríguez F. (2008). Estimation of ternary liquid–liquid equilibria for arene/alkane/ionic liquid mixtures using neural networks. Phys. Chem. Chem. Phys..

[23] Bhatti M.S., Kapoor D., Kalia R.K., Reddy A.S., Thukral A.K. (2011). RSM and ANN modeling for electrocoagulation of copper from simulated wastewater: Multi objective optimization using genetic algorithm approach. Desalination.

[24] Zhao G., Wang H., Liu G., Wang Z. (2016). Optimization of Stripping Voltammetric Sensor by a Back Propagation Artificial Neural Network for the Accurate Determination of Pb(II) in the Presence of Cd(II). Sensors.

[25] Suah F.B.M., Ahmad M., Taib M.N. (2003). Optimisation of the range of an optical fibre pH sensor using feed-forward artificial neural network. Sens. Actuators B Chem..

[26] Bade R., Bijlsma L., Miller T.H., Barron L.P., Sancho J.V., Hernández F. (2015). Suspect screening of large numbers of emerging contaminants in environmental waters using artificial neural networks for chromatographic retention time prediction and high resolution mass spectrometry data analysis. Sci. Total Environ..

[27] Torrecilla J.S., García J., Rojo E., Rodríguez F. (2009). Estimation of toxicity of ionic liquids in Leukemia Rat Cell Line and Acetylcholinesterase enzyme by principal component analysis, neural networks and multiple lineal regressions. J. Hazard. Mater..

[28] Alharbi N., Hassani H. (2016). A new approach for selecting the number of the eigenvalues in singular spectrum analysis. J. Frankl. Inst..

[29] Suykens J., Lukas L., Van Dooren P., De Moor B., Vandewalle J. Least squares support vector machine classifiers: A large scale algorithm. Proceedings of the European Conference on Circuit Theory and Design, ECCTD.

[30] Long B., Huang J., Tian S. Least squares support vector machine based analog-circuit fault diagnosis using wavelet transform as preprocessor. Proceedings of the International Conference on Communications, Circuits and Systems.

[31] Lin W.-M., Tu C.-S., Yang R.-F., Tsai M.-T. (2016). Particle Swarm Optimisation Aided Least-Square Support Vector Machine for Load Forecast with Spikes. IET Gener. Transm. Distrib..

[32] Zhang X., Wang J., Zhang K. (2017). Short-term electric load forecasting based on singular spectrum analysis and support vector machine optimized by Cuckoo search algorithm. Electr. Power Syst. Res..

[33] Pan A., Zhou J., Zhang P., Lin S., Tang J. (2017). Predicting of Power Quality Steady State Index Based on Chaotic Theory Using Least Squares Support Vector Machine. Power.

[34] Bian Q., Wang X., Xie R., Li T., Ma T. Small scale helicopter system identification based on Modified Particle Swarm Optimization, Guidance. Proceedings of the 2016 IEEE Chinese Navigation and Control Conference (CGNCC).

[35] Lagos-Eulogio P., Seck-Tuoh-Mora J.C., Hernandez-Romero N., Medina-Marin J. (2017). A new design method for adaptive IIR system identification using hybrid CPSO and DE. Nonlinear Dyn..

[36] Chuanwen J., Bompard E. (2005). A hybrid method of chaotic particle swarm optimization and linear interior for reactive power optimisation. Math. Comput. Simul..

[37] Tao H.Y.C. (2012). Phase-space reconstruction technology of chaotic attractor based on CC method. J. Electron. Meas. Instrum..

[38] Liu F., Liu F., Wang W., Xu B. MEMS gyro’s output signal de-noising based on wavelet analysis. Proceedings of the International Conference on Mechatronics and Automation.

[39] Kim H.S., Eykholt R., Salas J. (1999). Nonlinear dynamics, delay times, and embedding windows. Phys. D Nonlinear Phenom..

